# Deciphering age-specific molecular features in cervical cancer and constructing an angio-immune prognostic model

**DOI:** 10.1097/MD.0000000000037717

**Published:** 2024-04-12

**Authors:** Xin Zhao, Xichen Fan, Xiu Lin, Baozhu Guo, Yanqin Yu

**Affiliations:** a Department of Public Health, International College, Krirk University, Bangkok, Thailand; b Department of Oncology, Shengjing Hospital of China Medical University, Shenyang, China.

**Keywords:** age, cervical cancer, gene expression, immune, prognosis

## Abstract

Cancer incidence is increasingly seen in younger individuals. Molecular distinctions between young and elderly patients at onset are understudied. This study used public databases to explore genomic, transcriptomic, and immune-related features across age groups in cervical cancer. Additionally, it aims to create a prognostic model applicable across diverse age cohorts, enabling precise patient stratification, and personalized therapies. Gene mutations, expression data, and clinicopathological information were obtained from 317 cervical cancer patients. These patients were divided into a young group and an old group based on the median age of onset. The characteristics of differential gene mutation, gene expression, and immune cells analysis were analyzed by R software. Finally, the prognostic model was constructed by univariate Cox, least absolute shrinkage and selection operator, and multivariate Cox regression analyses of angiogenic and immune gene sets. Its validity was further confirmed using an additional 300 cervical squamous cell carcinoma and endocervical adenocarcinoma tissues. Cervical cancer patients at elderly onset age exhibit a significantly higher frequency of NOTCH1 and TP53 driver mutations compared to young patients, along with a notably higher tumor mutational burden. However, there were no significant differences between the 2 groups in terms of genomic instability and age-related mutational signatures. Differential gene expression analysis revealed that the young group significantly upregulated interferon-alpha and gamma responses and exhibited significantly higher activity in multiple metabolic pathways. Immune microenvironment analysis indicated enrichment of dendritic cells and natural killer cells in the young group, while transforming growth factor-β signature was enriched in the elderly group, indicating a higher degree of immune exclusion. A multigene prognostic model based on angiogenesis and T cell immune gene sets showed excellent prognostic performance independent of clinical factors such as age. High-risk groups identified by the model exhibit significant activation of tumor-promoting processes, such as metastasis and angiogenesis. Our study reveals distinct patterns in cancer-driving mechanisms, biological processes, and immune system status between young and elderly patients at onset with cervical cancer. These findings shed light on the age-specific underlying mechanisms of carcinogenesis. Furthermore, an independent molecular prognostic model is constructed to provide valuable references for patient stratification and the development of potential drug targets.

## 1. Introduction

Cervical cancer is a significant global health burden, consistently ranked as the third most prevalent cancer among females over the past decade. Despite advancements in screening and treatment, it remains the leading cause of cancer-related mortality among women in undeveloped countries.^[1]^ Age has been recognized as a pivotal factor influencing the prognosis and treatment outcomes of cervical cancer patients. The estimated incidence of cervical cancer varies widely among countries, with a global age-related incidence rate of 13.1 per 100,000 women.^[1]^ Notably, the disease’s biological behavior and response to therapies have been found to vary significantly across different age groups, prompting researchers to investigate the underlying mechanisms and develop tailored treatment strategies for each cohort.^[2]^

The underlying genetic predisposition to the outcome may vary with age and other risk factors, reflecting the underlying molecular mechanisms shaping the onset distribution.^[3,4]^ Younger women diagnosed with cervical cancer often exhibit distinct clinical characteristics and experience more aggressive disease courses. An epidemiologic and clinical analysis of cervical cancer from Japan showed that young patients in the radiotherapy group had a worse prognosis.^[5]^ Another case-control study demonstrated that certain genetic polymorphisms are the risk factor and can significantly reduce the risk of younger patients (≤49).^[6]^ However, a retrospective study provided data showing that the elderly population has much lower compliance and completion rates with surgery, RT, or chemotherapy as recommended standard practices.^[7]^ Therefore, understanding the pathogenic differences among age groups is essential for optimizing treatment approaches and improving overall survival rates.

Given that clinical prognosis still exhibits significant variability and is challenging to predict,^[8–10]^ more and more studies have been conducted to explore the biomarkers for the diagnosis and prognosis of cervical cancer. A study has revealed that, compared to the younger group, elderly patients (≥65) with cervical squamous carcinoma have a higher frequency of PIK3CA mutations. Moreover, these mutations are considered potential prognostic markers in the old populations.^[11]^ Another recent study has reported that a high level of TMEM33 expression can independently predict the prognosis of cervical cancer and correlate negatively with regulatory T cells and mast cells.^[12]^ However, comprehensive molecular characteristics and prognostic markers for patients in different age groups have yet to be fully elucidated.

In this study, we aim to explore the distinct carcinogenic characteristics of cervical cancer patients at different onset ages and establish a general prognostic model. Through a comprehensive analysis of genomic and transcriptomic differences between younger and elderly onset groups, we seek to elucidate molecular markers driving cancer initiation, progression, and treatment response in 2 age groups. The insights gained from this study will contribute to the progress of personalized medicine in the management of cervical cancer, ultimately enhancing survival rates and the quality of life for patients of all age ranges.

## 2. Methods

### 2.1. Data collection and age grouping of cervical cancer

RNA-sequencing data, somatic mutation data, and clinical information of 317 cervical cancer patients, were downloaded from the University of California, Santa Cruz Xena website (https://gdc.xenahubs.net). Using “cervical cancer” as the keyword, we downloaded a gene chip dataset GSE44001 containing 300 cervical squamous cell carcinoma and endocervical adenocarcinoma (CESC) tissues from the GEO database (https://www.ncbi.nlm.nih.gov/geo/). For The Cancer Genome Atlas (TCGA)-CESC, we retained samples with clinical information, survival time >0 and status (survival and death). For the GSE44001 dataset, we retained samples with complete clinical information and survival status. According to the density distribution of patient ages, the median age of 45 years was selected as the threshold to divide the patients into 2 groups: the Young Group and the Elderly Group, which were used for subsequent analyses.

### 2.2. Genomic feature calculation

Nonsynonymous single nucleotide variants and indels detected from whole exome sequencing were employed for calculating the tumor mutation burden by tallying the number of selected mutations and dividing it by the size of the whole exome sequencing region. The “deconstructSigs” R package was utilized to determine the contribution of known mutational processes to a tumor sample.^[13]^ The reference mutational signatures were obtained from the Catalogue of Somatic Mutations in Cancer, with single base substitution classes identified using 96 different contexts. Additional data, including loss of heterozygosity (LOH), ploidy, and the number of whole genome doublings, were gathered from a previous study.^[14]^ Here, tumor purity and genomic instability indices in TCGA tumor samples were estimated by the ABSOLUTE algorithm, which is based on copy number and mutation data.

### 2.3. Differential expression analysis

For expression analysis, only fresh tumor tissue samples obtained from the primary tumor site were retained. In cases where multiple expression data were available for the same gene, the maximum value was selected. The DESeq2 R package was utilized to test for differential expression based on read counts.^[15]^ Genes with adjusted *P* values <.05 were defined as differentially expressed between groups. Among these genes, those with a fold change >2 were considered significantly upregulated, while those with a fold change <0.5 were considered significantly downregulated. The functional interaction network of differential genes was constructed by GeneMANIA, available at https://genemania.org/.

### 2.4. Pathway enrichment analysis

Gene set enrichment analysis (GSEA) was conducted by the “clusterProfiler” R package^[5]^ (v3.18.1) with the gene set collections h.all.v7.4.entrez.gmt and c2.cp.kegg.v7.4.entrez.gmt from MSigDB.^[16]^ The top KEGG or hallmark terms were displayed when the adjusted *P* value, determined using the Benjamini–Hochberg method, was <.05, indicating statistical significance. The immuno-oncology biological research R package^[17]^ was used to calculate scores for oncogenic pathways, metabolic pathways, or immune-related features in each sample based on the single-sample GSEA (ssGSEA) method.

### 2.5. Composition of tumor-infiltrating immune cells

Multiple computational tools were employed to estimate the abundances of tissue-infiltrating immune and stromal cell populations based on gene expression profiles. These tools include CIBERSORT,^[18]^ MCPCounter,^[19]^ and TIMER.^[19]^ Immunological exclusion features were collected from previous studies.

### 2.6. Survival analysis

Kaplan–Meier curves were generated to illustrate the difference in overall survival between the 2 groups. Univariate Cox regression analysis was performed to identify survival-related genes. Multivariable Cox regression analysis was performed to assess the independence of each prognostic factor. All the aforementioned analyses were carried out using the R packages “survival” and “survminer.”

### 2.7. Construction of the prognostic risk score model

After obtaining survival-related angiogenesis and T cell immunity (AT) genes, the least absolute shrinkage and selection operator (LASSO) Cox regression analysis was employed to construct the AT-related prognostic model by the “glmnet” and ‘survival’ packages. The penalty parameter (λ) was determined using the minimum criteria. Based on the results of the LASSO regression analysis, a risk score for each patient was calculated using the expression levels of genes and their corresponding coefficients in the prognostic model. The formula used for calculating the risk score was as follows: Riskscore = (Exp_gene1_ × Coef_gene1_) + … + (Exp_gene n_ × Coef_gene n_), where “Coef” represents the coefficient and “Exp” indicates the expression level of genes. TCGA-CESC patients were divided into 2 subgroups (high-risk and low-risk) based on the median risk score. Receiver operating characteristic analysis was used to evaluate the accuracy of our model, which was conducted using the “survival,” “survminer,” and “timeROC” packages.

### 2.8. Statistical analysis

All statistical analyses were performed using R software (version 4.0.3, https://www.r-project.org/). Differences between 2 groups were assessed using Fisher exact test for categorical variables and Wilcoxon test for continuous variables. The log-rank test was employed to assess differences in multiple survival metrics between groups. A *P* value of <.05 was considered statistically significant.

## 3. Results

### 3.1. Gene mutation profiles

**Figure 1.**
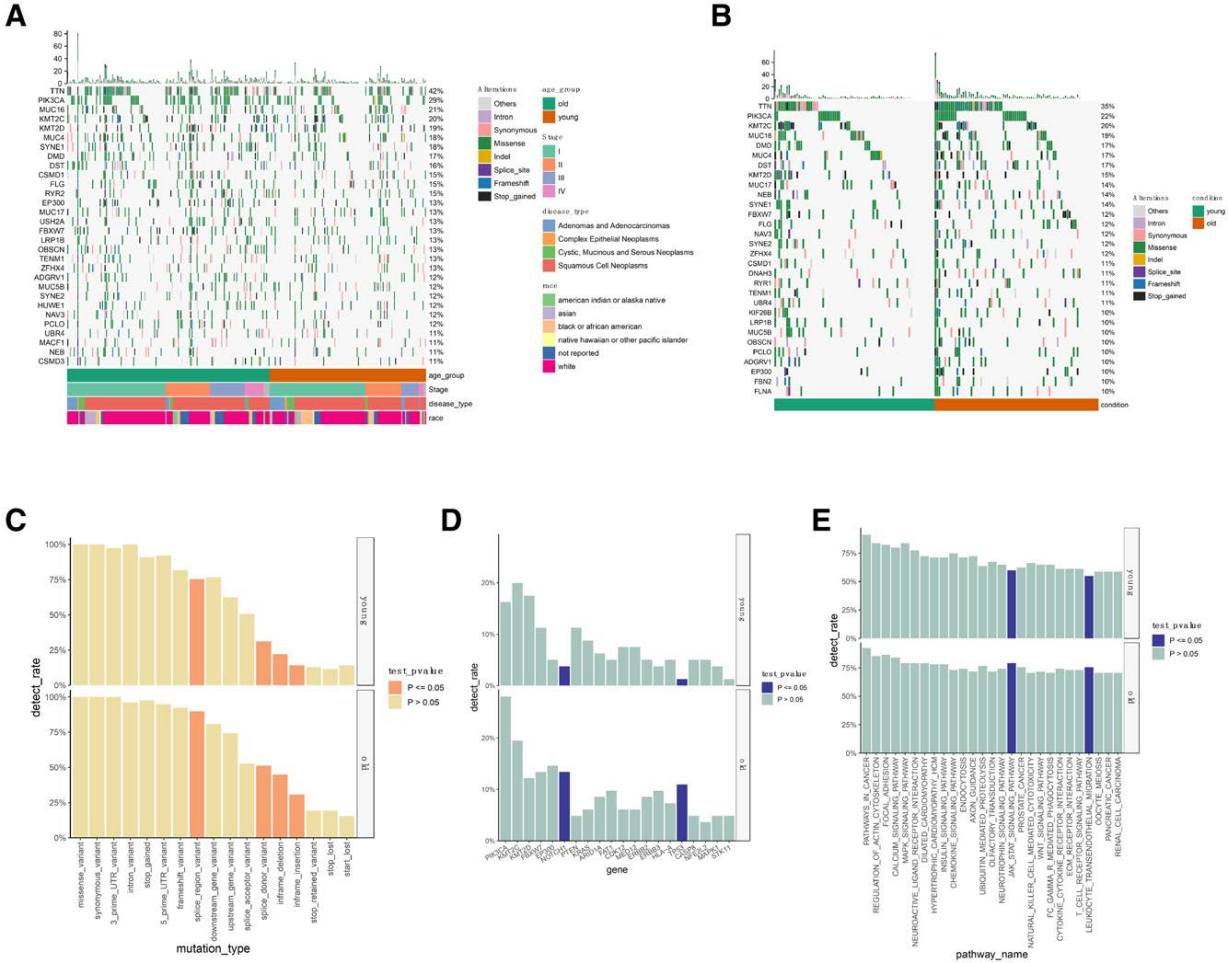
Genomic landscape of cervical cancer patients. Somatic mutation profile of all cervical cancer patients (A) and stratified for young and elderly groups among stage I patients (B). Different colors in the heatmap represent the types of mutations. Clinical information groups are depicted below the heatmap. Comparison of mutation detection rates between young and elderly groups for mutation types (C), driver genes (D), and mutation pathways (E), with significantly different terms marked by dark bars, and vice versa by light bars.

The complete process of constructing cervical cancer prognosis model in this study is shown in Figure S1, Supplemental Digital Content, http://links.lww.com/MD/M43. After analyzing the mutation spectrum across all samples, several genes were found to have high-frequency mutations in cervical cancer. Notably, TTN, PIK3CA, MUC16, KMT2C/D, MUC4, and SYNE1 were identified as the genes with the most prevalent mutations. Among them, TTN exhibited the highest mutation frequency, occurring in 42% of the patients, closely followed by PIK3CA with a mutation frequency of 29% (Fig. 1A). Upon closer examination, the PIK3CA gene emerged as one of the most significantly mutated genes, featuring 2 prominent mutation sites, p.E545K and p.E542K (Table S1, Supplemental Digital Content, http://links.lww.com/MD/M39). These specific mutations have been implicated in various malignancies, emphasizing their importance in the context of cervical cancer pathogenesis.

In order to investigate the distinct cancer driver mechanisms between the young and elderly groups, we conducted a comparative analysis of gene mutation profiles involving 162 patients, including 80 individuals in the young group and 82 individuals in the elderly group. All patients were in stage I, aiming to exclude the confounding effects of molecular features associated with different cancer stages. The high-frequency mutation genes in this cohort, namely TTN, PIK3CA, KMT2C, and MUC16, were consistent with those in the general population (Fig. 1B). We compared the mutation types between the 2 groups and found a significant enrichment of splice site mutations and inframe insertions/deletions in the elderly group (Fig. 1C).

Driver gene mutations are generally considered to confer a selective growth advantage in the development of cancer compared to passenger mutations, which are generally thought to have no functional consequences. We acquired a list of cancer driver genes specific to CESC from the IntOGen-Cancer Mutations Browser (https://www.intogen.org/) and conducted a comparison of the mutation frequencies of these driver genes between the 2 age groups. We noted a significantly higher frequency of NOTCH1 and TP53 mutations in the elderly group (Fig. 1D), a correlation also observed in Childhood Malignant Gliomas.^[20]^

These results indicate a distinct molecular pathogenetic basis between young and elderly CESC patients with similar lesions. Furthermore, upon integrating mutations into biological pathways, we found a significantly higher mutation rate in the JAK/STAT signaling pathway and leukocyte transendothelial migration pathway in the elderly group (Fig. 1E).

### 3.2. Differential genomic features between young and elderly groups

**Figure 2.**
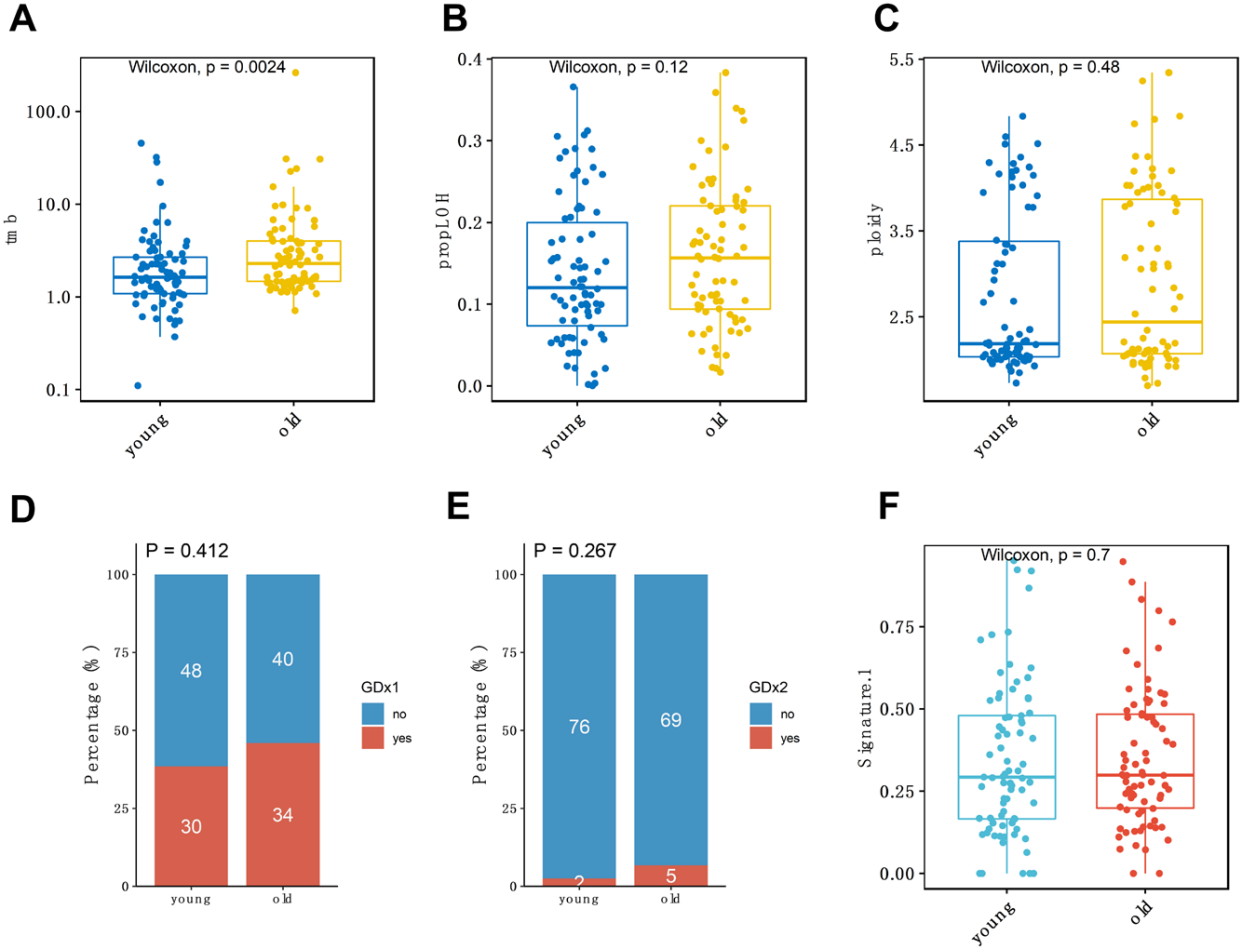
Comparison of genomic features between young and elderly groups. Comparison of tumor mutation burden (A), LOH proportion (B), and ploidy (C) between young and elderly groups, with *P* values obtained from Wilcoxon tests. Proportion of patients with whole genome duplications at 1× (D) and 2× (E) within each age group, with *P* values obtained from Fisher tests. (F) Distribution of COSMIC mutational signature 1 between the 2 groups, with *P* values from Wilcoxon tests. COSMIC = Catalogue of Somatic Mutations in Cancer, LOH = loss of heterozygosity.

To assess the differences in global genomic features at the onset of cancer between different age groups, we initially compared the tumor mutation burden between the 2 groups. We found that even though both groups had tumors in the early stage, the elderly group tended to accumulate more mutations in the genome, exhibiting a significantly higher tumor mutational burden (Fig. 2A).

Previous studies have suggested a correlation between age and genomic instability, but this correlation is cancer-specific and has led to contradictory conclusions.^[21,22]^ In this study, we aimed to specifically investigate whether CESC patients of different ages at the early stages exhibit differences in genomic instability, which could contribute to distinct carcinogenic pathways. LOH is an allelic imbalance event in which a heterozygous somatic cell becomes homozygous because one of the 2 alleles is lost. A previous research has suggested that LOH may generate cancer-specific vulnerabilities and serve as potential therapeutic targets in cancer.^[23]^ The ploidy of cancer cells refers to the amount of DNA they contain, with the typical amount in normal cells being referred to as diploid. Ploidy changes are usually closely associated with chromosomal instability and play a role in tumor initiation and cancer progression.^[24–26]^ We found that the LOH ratio and ploidy did not significantly differ between the young and elderly groups but showed an increasing trend in the elderly group (Fig. 2, B and C).

“Whole genome doubling” (WGD), where the entire set of chromosomes in a cell is duplicated, is another genomic event that promotes chromosomal instability and the acquisition of aneuploidies.^[27]^ We observed that 38.5% (30/78) of patients in the young group and 45.9% (34/74) of patients in the elderly group had WGD events. Among them, 2 patients in the young group and 5 patients in the elderly group had their whole genome copy number doubled, indicating that the elderly group exhibited a slightly higher incidence of WGD events compared to the young group (Fig. 2, D and E).

We then leveraged the Catalogue of Somatic Mutations in Cancer mutational signatures to deconvolve mutational processes in each patient. We observed mutational accumulations in Signature SBS1 (clock-like signature), SBS7 (UV light exposure), SBS2, and SBS13 (APOBEC activity), but none of these signatures showed significant differences between the age groups (Figure S2A–C, Supplemental Digital Content, http://links.lww.com/MD/M44, Table S2, Supplemental Digital Content, http://links.lww.com/MD/M40). Interestingly, the number of mutations related to Signature SBS1 correlated with the age of the individual, but there was no difference between our young and elderly groups (Fig. 2F). This suggests that the higher mutation load observed in the elderly group is not solely due to age but may be related to the specific mutational contexts required for carcinogenesis in different age groups.

### 3.3. Age-specific transcriptional signatures

**Figure 3.**
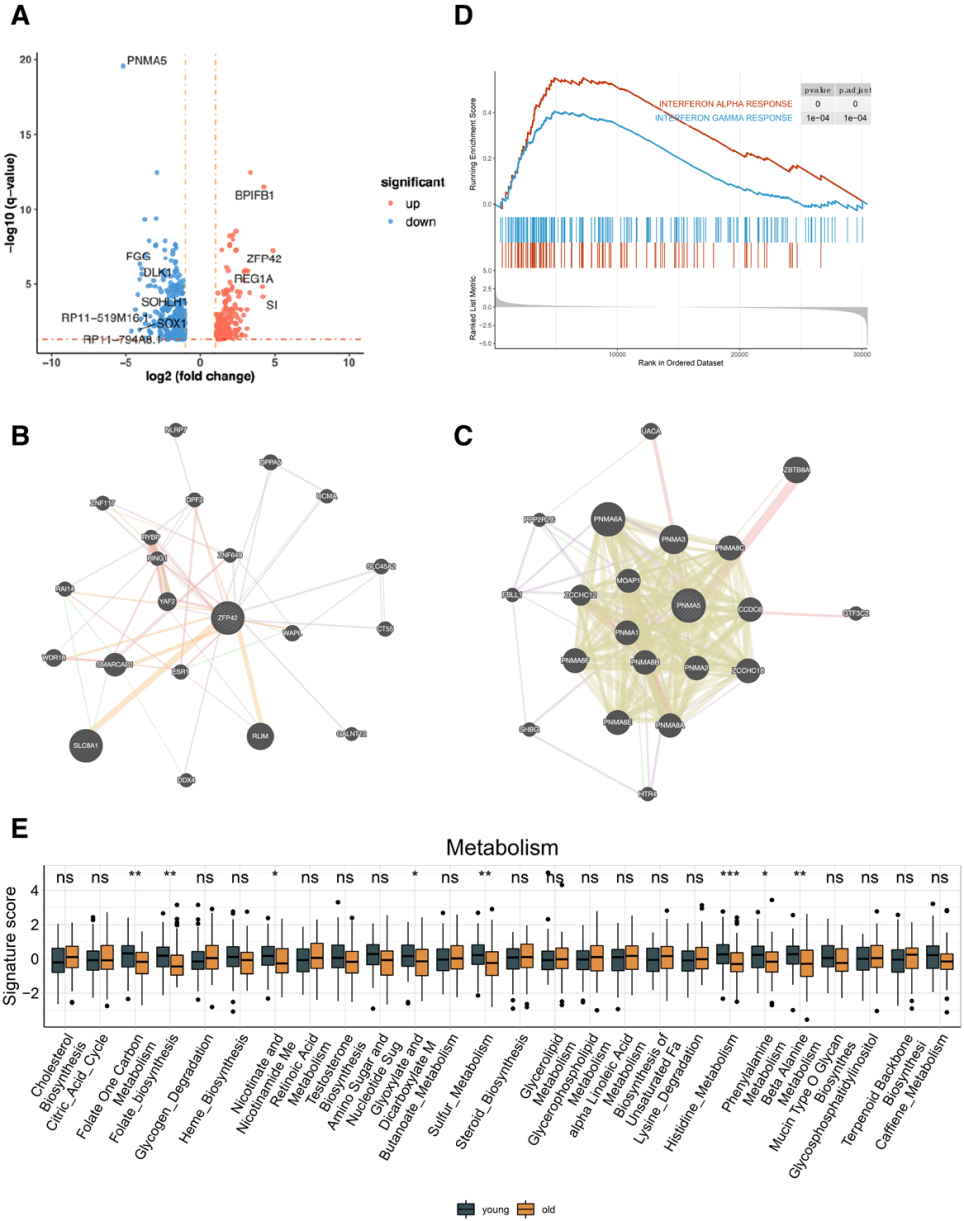
Age-specific gene and pathway expression. (A) Genes significantly upregulated (red dots) and downregulated (blue dots) in the young group compared to the old group. The horizontal line represents the threshold with a corrected *P* value of .05, and the vertical lines represent a log2 (fold change) >1 or <−1, with labels indicating genes with the greatest differences having a log2 (fold change) >4 or <−4. The functional interaction network of ZFP42 (B) and PNMA5 (C). (D) GSEA plot displaying pathways significantly upregulated in the young group. (E) Differences in the activity of various metabolic pathways between the young and old groups, assessed for significance using the Wilcoxon test, where *P < .5, **P < .01, ***P < .001. GSEA = gene set enrichment analysis, ns = no significant difference.

Age-related transcriptional programs have been observed in various cancer types to date, including extracellular matrix organization, metabolism, and distinct signaling pathways.^[28–30]^ We initially computed age-related differentially expressed genes to reflect the transcriptional differences at cancer onset in individuals of different ages and to identify unique molecular markers in different age groups. The results revealed that there were 665 differentially expressed genes between the young and elderly groups, of which 220 genes were upregulated and 445 genes were downregulated in the young group compared to the elderly group (Fig. 3A).

Among the most significantly upregulated genes in the young group was ZFP42, a zinc finger transcription factor and a well-known stem-cell marker (Fig. 3B). ZFP42 encodes the Rex1 protein, which activates the MEK/ERK pathway to promote tumorigenesis in prostate cancer and promotes EMT-induced cell metastasis by activating the JAK2/STAT3-signaling pathway in cervical cancer.^[31,32]^ Conversely, the most significantly downregulated gene in the young group was PNMA5, which encodes a member of the paraneoplastic Ma antigen protein family (Fig. 3C). PNMA5 has an oncogenic effect in human breast cancer and cervical cancer cell lines, and studies have indicated its role in promoting bone metastasis of non-small-cell lung cancer.^[33,34]^

GSEA identified significant activation of interferon-alpha (IFN-α) and gamma (IFN-γ) responses within the young group (Fig. 3D), which is associated with innate and adaptive immunity. Additionally, we employed single-sample GSEA based on gene expression data to compute the expression activity of multiple tumor-related features. Interestingly, we found that there were no significant differences between the 2 groups in features related to the cell cycle, DNA damage repair, or hypoxia (Figure S2D, Supplemental Digital Content, http://links.lww.com/MD/M44). However, the young group exhibited significantly higher activity in various metabolic pathways. This includes pathways involved in Folate One Carbon Metabolism, Folate biosynthesis, Sulfur Metabolism, and Histidine Metabolism, among others (Fig. 3E). These findings suggest that the young group may have a more active metabolic profile compared to the elderly group, potentially reflecting distinct metabolic adaptations in different age groups in the context of cervical cancer.

### 3.4. Comparison of immune microenvironment between different age groups

**Figure 4.**
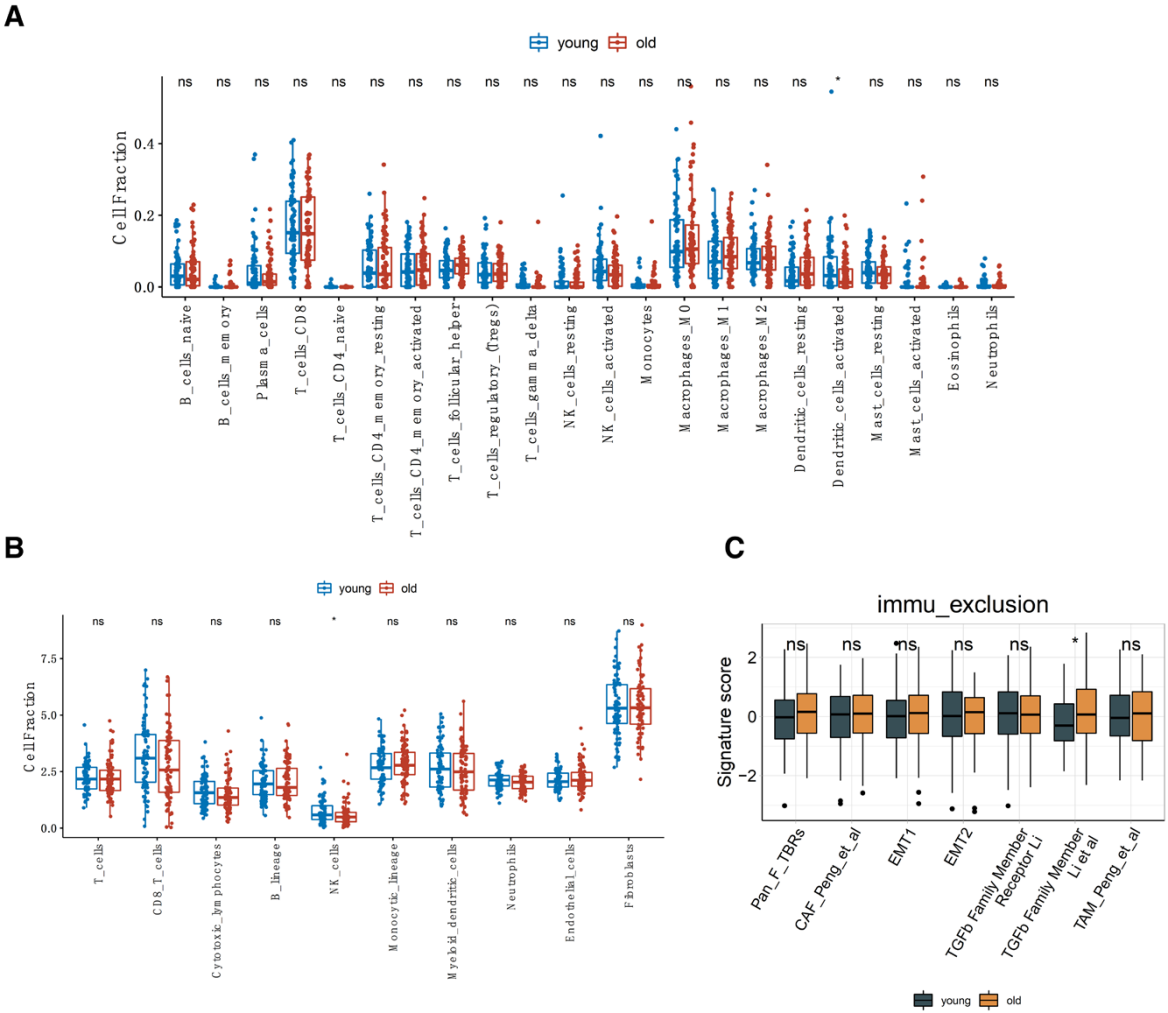
Differences in the immune microenvironment between the young and elderly groups. Immune cell composition differences assessed through CIBERSORT (A) and MCPCounter (B). (C) Differences in the activity scores of various immune exclusion signatures collected from published literature between the 2 groups. All comparisons were conducted using the Wilcoxon test. *P < .5. ns = no significant difference.

The proportions of 22 immune cell types were obtained by deconvoluting bulk expression matrices using CIBERSORT. We found that the majority of these cell types showed no significant differences in abundance between the young and elderly groups, except for activated dendritic cells, which were significantly higher in the young group (Fig. 4A). Furthermore, when estimating 10 cell types using MCPCounter, we observed that only natural killer (NK) cells were significantly higher in the young group compared to the elderly group (Fig. 4B).

These 2 cell types are primary components of the innate immune system and play crucial roles as mediators of effective immune responses against infections and diseases.^[35]^ The reciprocal interaction between dendritic cells and NK cells results in activating cross-talk. For example, mature dendritic cells can activate resting NK cells, leading to the secretion of IFN-γ by NK cells and enhancing their cytotoxic activity.^[36]^ In contrast, activated NK cells can induce dendritic cell maturation. Furthermore, coordinated mechanisms between dendritic cells and NK cells are vital for initiating and coordinating adaptive immunity in cancer.^[37]^

These results suggest the activation of innate immunity in the immune microenvironment of the young group, consistent with the earlier observation of upregulation of the IFN-γ pathway in the young group. On the other hand, when calculating the enrichment scores for 7 immune exclusion features in the 2 groups, we found that transforming growth factor-β (TGF-β) signature was enriched in the elderly group, indicating a higher degree of immune exclusion in the elderly group compared to the young group (Fig. 4C).

### 3.5. Age-independent angio-immune prognostic model

We observed that age grouping alone did not directly differentiate overall survival in cervical cancer patients, even for stage I patients (Figure S3A–B, Supplemental Digital Content, http://links.lww.com/MD/M45). A total of 3721 survival-related genes were identified through univariate Cox regression analysis in the entire population. To investigate whether age affects the selection of prognostic genes, we separately identified prognosis-related genes in the young and elderly groups. We found that there is a low overlap of prognosis-related genes between the 2 age groups, and a significant proportion of these genes are age-specific in terms of prognosis (Figure S3C–D, Supplemental Digital Content, http://links.lww.com/MD/M45).

To obtain a molecular prognostic indicator independent of age and other clinical factors, we constructed a prognostic model based on recently published pan-cancer immune subtypes related to AT. Studies have revealed that AT activity can be used to stratify the tumor microenvironment and predict the therapeutic benefits of immune checkpoint blockade treatment in patients.^[38]^ Therefore, the AT genes are believed to comprehensively reflect the tumor status and predict patient prognosis.

**Figure 5.**
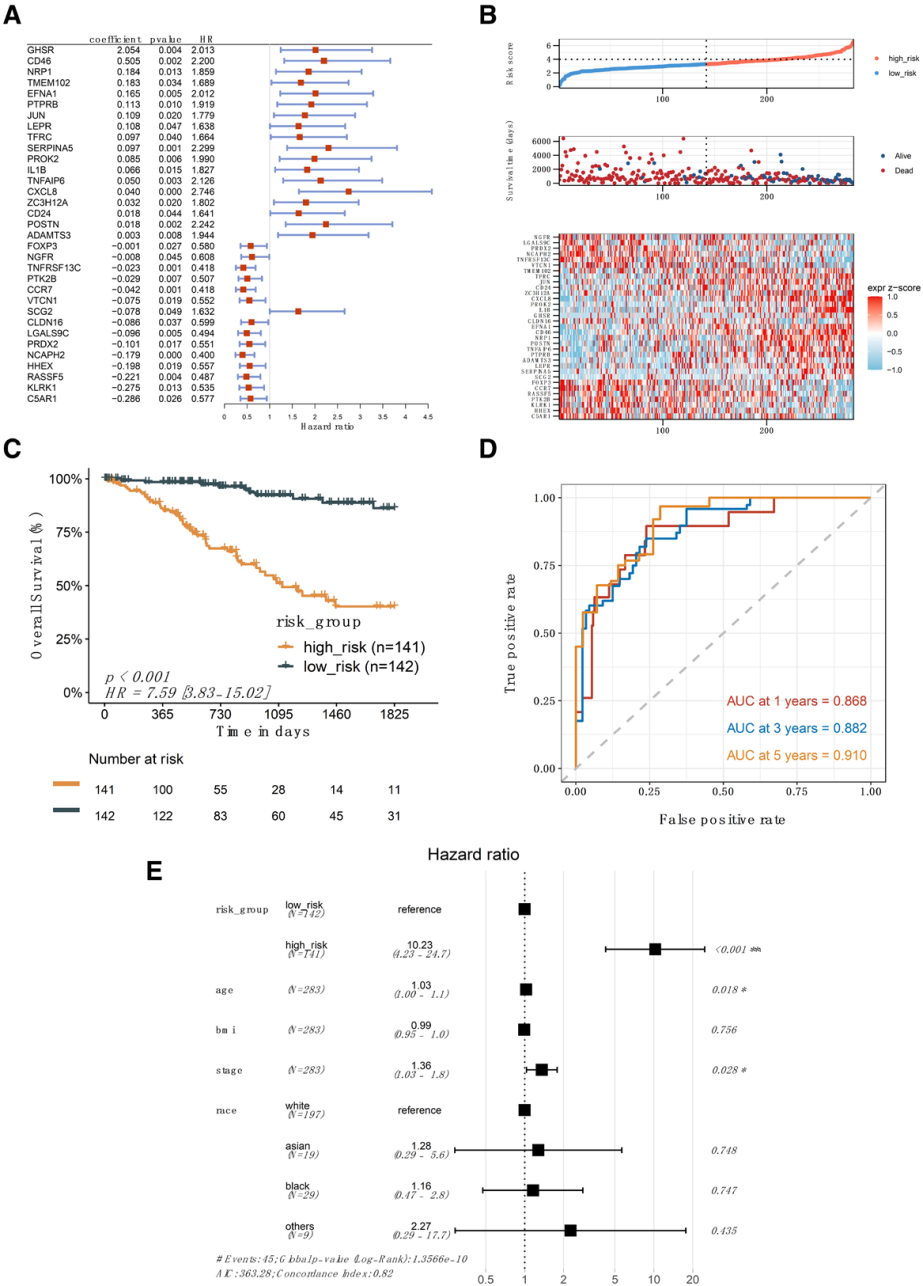
Construction and performance evaluation of the angio-immune prognostic model. (A) The 33-gene signature identified through LASSO-Cox regression analysis. It displays the coefficients for each gene, as well as the *P* values and hazard ratios from univariate regression analysis. (B) Distribution of risk scores calculated by the model, scatter plots of survival time, and a heatmap illustrating the expression of the 33 genes in cervical cancer samples. (C) Survival curves comparing high and low-risk groups, with *P* values obtained from log-rank tests. (D) ROC analysis evaluating the predictive performance of the risk model. (E) Results of the multivariate Cox regression analysis, displaying hazard ratios and *P* values for each included factor. LASSO = loss of heterozygosity, ROC = receiver operating characteristic.

A total of 1151 AT genes were initially collected from the literature (Table S3, Supplemental Digital Content, http://links.lww.com/MD/M41), and after removing noncoding genes, 1047 genes remained. Among them, 152 genes were identified through univariate Cox regression analysis to be significantly associated with prognosis (Table S4, http://links.lww.com/MD/M42). These genes were used to construct a multigene prognostic model. Gene expression and survival data were extracted from tumor samples of 283 patients in TCGA. Using LASSO-Cox regression analysis, a prognostic model consisting of 33 genes was selected (Fig. 5A). Multiplying the expression level of each gene by its corresponding coefficient and summing them up yields the risk score value of the model. Based on the median risk score values derived from the model, patients were categorized into 2 groups: high-risk and low-risk.

The expression levels of each model gene, risk score, and survival status for each patient are shown in Figure 5B. It was observed that the high-risk group exhibited a significantly lower 5-year overall survival rate compared to the low-risk group (hazard ratio = 7.59, *P* < .001) (Fig. 5C). The model demonstrated strong prognostic performance with area under the ROC curve values of 0.868, 0.882, and 0.910 at 1, 3, and 5 years, respectively, indicating excellent predictive ability (Fig. 5D). Furthermore, when conducting multivariate regression analysis, considering clinical and demographic factors such as age, body mass index, stage, and race, the risk group was identified as an independent and significant prognostic factor (*P* < .001, Fig. 5E). We additionally used the GSE44001 dataset as an external validation of the model, and the results showed that there was a significant difference in survival between the high-risk group and the low-risk group (Figure S4, Supplemental Digital Content, http://links.lww.com/MD/M46). Currently, there are few high-throughput data sets for cervical cancer, and more and larger cohorts are needed to verify the performance of the model, and we will continue to pay attention to and track emerging high-throughput data sets.

### 3.6. Differential biological features between high and low-risk groups

**Figure 6.**
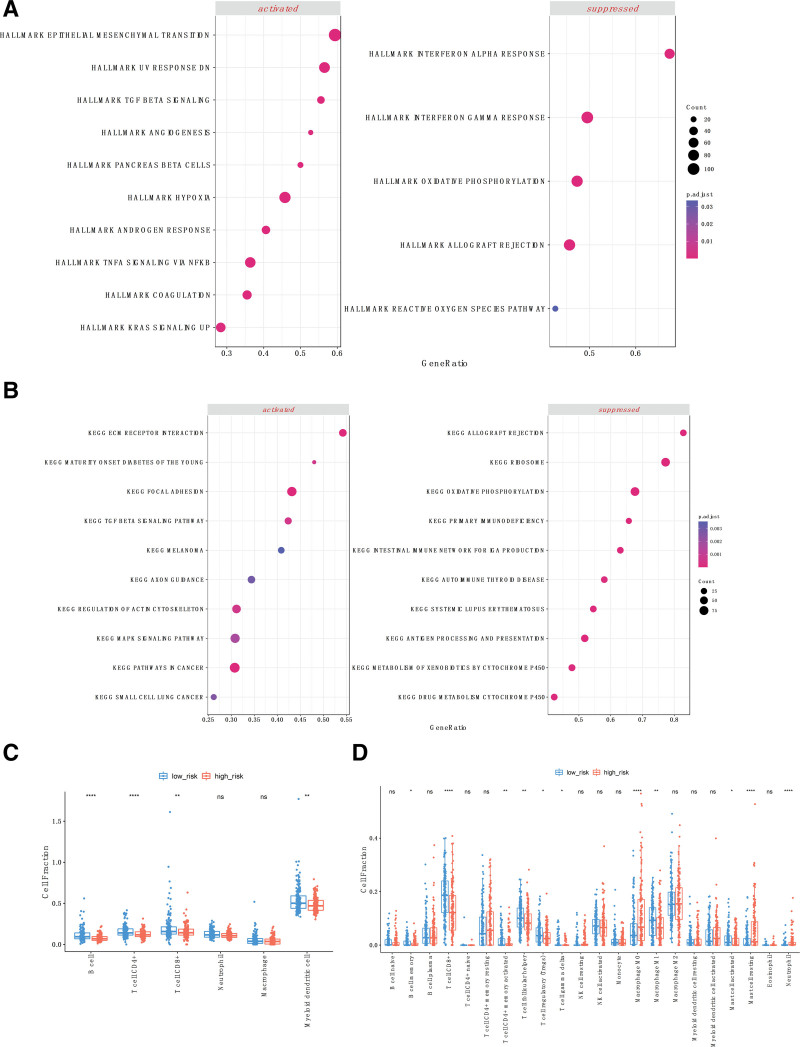
Biological feature differences between high and low-risk groups. Differential expression of cancer hallmarks (A) and KEGG pathways (B) between the high-risk and low-risk groups. The bubble plot on the left represents activated biological processes in the high-risk group, while the right side represents suppressed processes. Estimation of immune cell composition from TIMER (C) and CIBERSORT (D) showing differences between the 2 groups.

Differential expression pathways between the high-risk and low-risk groups were investigated through GSEA analysis. In the cancer hallmark gene set, the high-risk group exhibited significant upregulation of pathways related to epithelial-mesenchymal transition and angiogenesis, while suppressing immune pathways such as IFN-α and IFN-β responses, as well as oxidative phosphorylation (Fig. 6A). KEGG GSEA indicated an upregulation of extracellular matrix pathways and the mitogen-activated protein kinases signaling pathway in the high-risk group (Fig. 6B). Activation of the TGF-β signaling pathway was identified in both gene sets. Taken together, TGF-β induction of epithelial-mesenchymal transition, promoting tumor invasion and metastasis,^[39]^ along with its further activation of tumor angiogenesis and cancer-associated fibroblasts, enabling immune response evasion,^[40]^ may explain the poor prognosis observed in the high-risk group. Furthermore, we observed an enrichment of CD4 + and CD8 + T cells in the low-risk group, representing an activated antitumor immune environment. This finding is consistent with the results from TIMER and CIBERSORT analyses (Fig. 6, C and D).

## 4. Discussion

China is among the few countries facing a rising incidence of cervical cancer.^[41]^ As the age-related increase in morbidity and mortality has been widely recognized, research has also begun to focus on the connection between age and the intrinsic tumor biology in order to refine treatment approaches for different age groups and improve survival rates. Several studies have identified unique molecular features associated with age in different cancer types, including breast,^[28,37]^ colorectal,^[42]^ and prostate^[43]^ cancers. However, there is still a lack of systematic comparative studies on the multiomics characteristics of young and elderly onset cervical cancer. In the present study, we categorized patients into young and elderly groups based on the threshold of the median age of onset, which is 45 years, utilizing publicly available TCGA cohorts. We specifically selected stage I cervical cancer patients for analysis to eliminate progression-related changes in molecular characteristics resulting from different stages. We systematically characterized the differential landscape of the genome, transcriptome, and immune microenvironment in cervical cancer across different age groups. Additionally, to effectively guide prognosis stratification for cervical cancer patients and inform subsequent treatment selection, we developed a multigene prognostic model based on AT. This model can independently classify patients into significantly distinct high-risk and low-risk groups, irrespective of age.

The differences in genomic alterations between different age groups may indicate age-related carcinogenic mechanisms. First, we observed that the elderly group had a higher enrichment of splice site mutations and inframe insertions/deletions compared to the young group. These types of mutations may have a higher tendency to occur in tumor suppressor genes, disrupting their growth-inhibitory functions.^[44]^ Additionally, studies have shown that neoantigens derived from indel mutations were enriched for mutant-specific binding, as compared to neoantigens derived from nonsynonymous single nucleotide variant mutations.^[45]^ Furthermore, we found that the elderly group had a higher enrichment of NOTCH1 and TP53 mutations, both of which are considered important tumor suppressor genes in cervical cancer. In contrast, in colorectal and breast cancers, it has been reported that TP53 alterations were more common in younger patients. These results indicate that the differences in genomic-level mutations between different age groups may exhibit cancer-type specificity.

Consistent with previous researches indicating an increase in somatic mutation count with age,^[26,46,47]^ we observed a significantly higher tumor mutational burden in the elderly group in cervical cancer. However, in contrast to previous pan-cancer analyses that showed positive associations between age and genomic instability,^[29]^ we did not observe significant differences between the young and elderly groups in terms of LOH proportion, ploidy, and WGD. Furthermore, previous studies on age-related mutation signatures in breast cancer revealed a significant enrichment of homologous recombination deficiency signature S3 in younger patients.^[28]^ In our analysis, we also observed a statistically significant phenomenon of homologous recombination deficiency signature S3 enrichment in the young group compared to the elderly group (Wilcoxon test *P* = .034) (Table S2, Supplemental Digital Content, http://links.lww.com/MD/M40). However, this feature was only observed in a small subset of patients, suggesting that DNA repair deficiencies may be a stronger etiological factor in some patients in the younger group. Age-related signature S1, UV light exposure signature S7, APOBEC signatures S2 and S13 were the most predominant signatures identified, and there were no significant differences between the 2 groups regarding these signatures.

Based on transcriptomic data, we observed a significant upregulation of IFN-related pathways in the younger group, while concurrent analysis of the immune microenvironment revealed an enrichment of dendritic cells and NK cells in the younger group. These features collectively indicate the activation of innate immunity in the younger group. In contrast, the elderly group exhibited an enrichment of the TGF-β signature, consistent with previous research in breast cancer. However, in the case of Kidney Renal Papillary Cell Carcinoma, young adult cases showed elevated TGF-β response and proliferation,^[30]^ suggesting that the impact of age on innate and adaptive immunity varies across different tissues. Moreover, these age-related immune feature differences also have the potential to influence the suitability of immunotherapy for patients, such as anti-TGF-β therapies. Additionally, we also observed the activation of multiple metabolic pathways in the younger group. A previous research has suggested that metabolic dysregulation, such as that induced by obesity, may have contributed to the recent increase in cancer incidence among young adults.^[48]^ We speculate that exploring the use of drugs that target specific metabolic pathways may be a worthwhile direction to investigate for the treatment of young cervical cancer patients.

Differences in tumor characteristics between different age groups may lead to distinct prognostic outcomes. For example, breast cancer tends to exhibit a more aggressive nature and poorer survival rates in younger patients,^[49]^ whereas elderly patients with ovarian cancer generally have an unfavorable prognosis.^[50]^ In our current study, we did not observe differences in overall survival between the young and elderly groups. Therefore, we constructed a multigene prognostic model based on AT-related genes. The median risk score calculated by this model can be used to divide patients into high-risk and low-risk groups, independently distinguishing between individuals with different prognoses, regardless of factors such as age and stage. The high-risk group exhibited the activation of multiple pathways associated with metastasis, suggesting that this model may predict the metastatic potential in early-stage patients. Furthermore, considering the current use of immunotherapy and antiangiogenic therapy in first-line treatment for advanced cervical cancer, additional training, and optimization of the model with relevant treatment efficacy data may allow it to serve as a predictive biomarker guiding treatment strategies for late-stage patients.

In conclusion, this study, for the first time, provided a systematic exploration of key genomic, transcriptomic, and immune microenvironment features in cervical cancer patients across different age groups. The differential carcinogenic processes between these groups may provide insights for distinct therapeutic strategies and biomarker discovery. However, there are some limitations to this study. First, to ensure that both the younger and elderly groups were at a similar tumor stage, thus enabling a better reflection of the differences in carcinogenic mechanisms between the 2 groups, we limited our comparative analysis to stage I patients, which resulted in a reduced sample size. Second, the patients used for analysis were primarily of Caucasian ethnicity, and larger cohorts of Asian patients could provide more valuable clinical insights. Additionally, the clinical utility of the prognostic model needs validation in larger independent cohorts. Finally, integrating more omics data, including epigenomics and proteomics, and exploring the upstream-downstream coordination between multiple omics, warrant further mechanistic and clinical studies.

## Author contributions

**Conceptualization:** Xin Zhao.

**Data curation:** Xin Zhao, Xichen Fan.

**Formal analysis:** Xin Zhao, Xichen Fan.

**Investigation:** Xin Zhao, Yanqin Yu.

**Methodology:** Xin Zhao, Baozhu Guo.

**Project administration:** Xin Zhao.

**Resources:** Xin Zhao, Xiu Lin.

**Software:** Xin Zhao, Xiu Lin.

**Supervision:** Xin Zhao, Yanqin Yu.

**Validation:** Xin Zhao, Baozhu Guo.

**Visualization:** Xin Zhao.

**Writing—original draft:** Xin Zhao.

**Writing—review & editing:** Xin Zhao, Yanqin Yu.

## Supplementary Material

**Figure:**
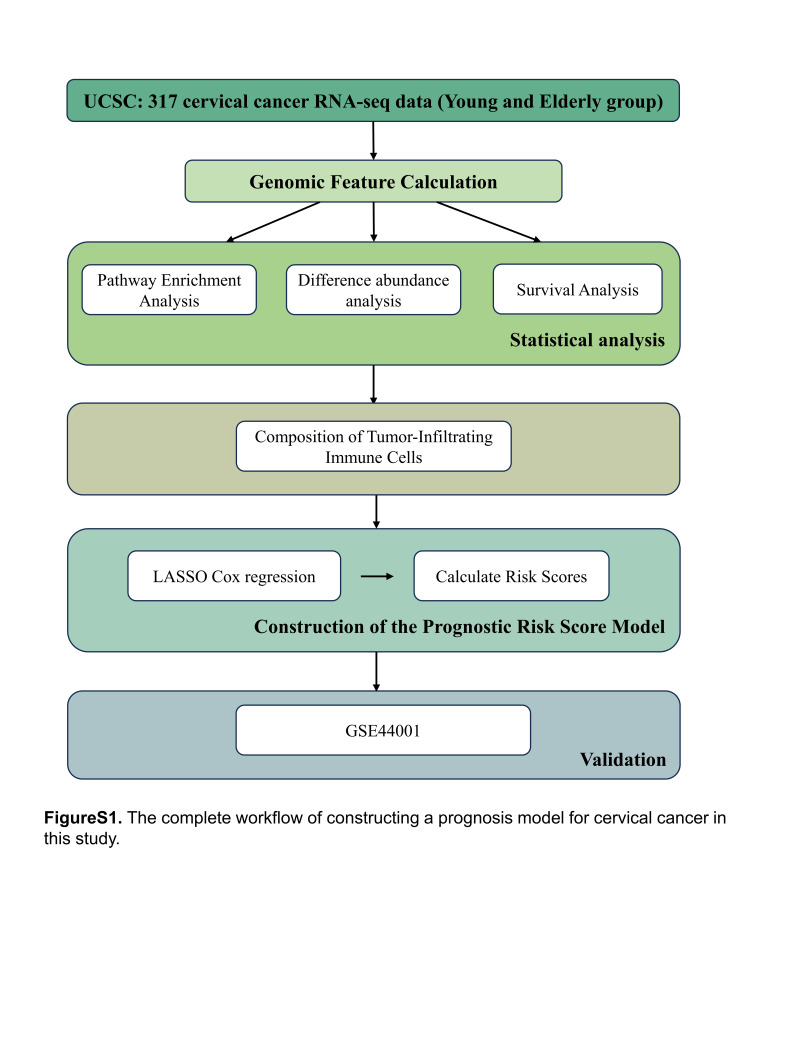


**Figure:**
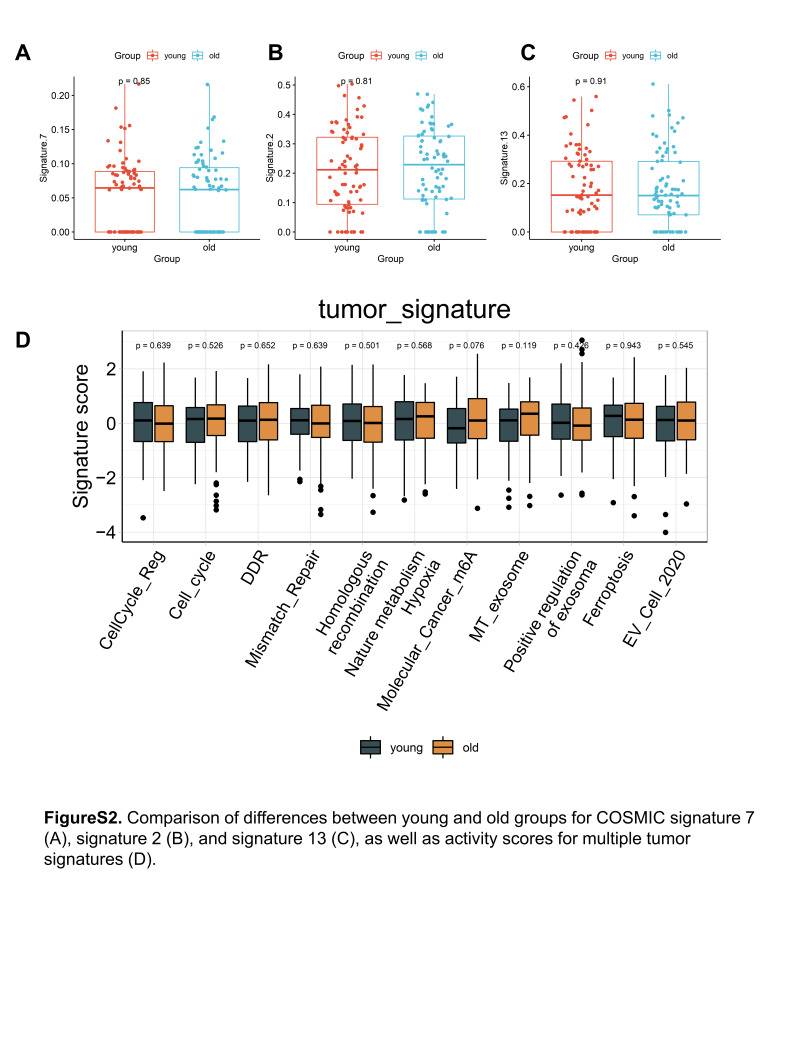


**Figure:**
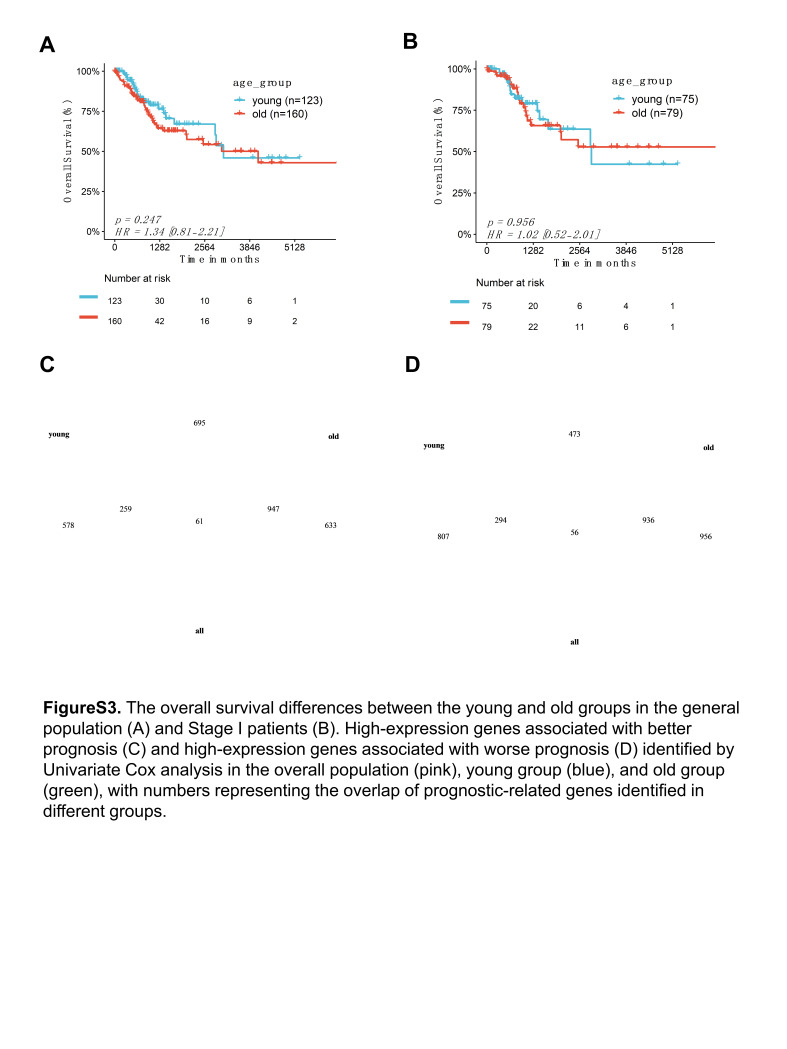


**Figure:**
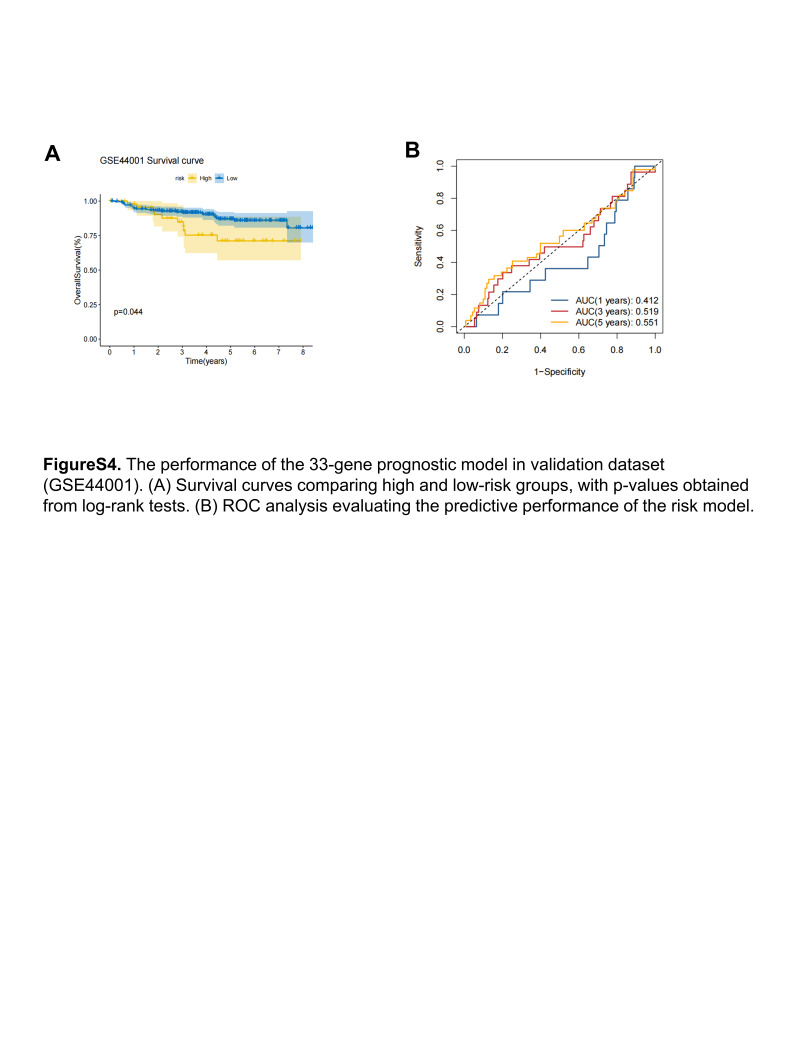










## References

[1] Arbyn M Weiderpass E Bruni L. Estimates of incidence and mortality of cervical cancer in 2018: a worldwide analysis. Lancet Glob Health. 2020;8:e191–203. 31812369 10.1016/S2214-109X(19)30482-6 PMC7025157

[2] Clarke MA Risley C Stewart MW. Age-specific prevalence of human papillomavirus and abnormal cytology at baseline in a diverse statewide prospective cohort of individuals undergoing cervical cancer screening in Mississippi. Cancer Med. 2021;10:8641–50. 34734483 10.1002/cam4.4340 PMC8633239

[3] Schiffman M Doorbar J Wentzensen N. Carcinogenic human papillomavirus infection. Nat Rev Dis Primers. 2016;2:16086. 27905473 10.1038/nrdp.2016.86

[4] Ojavee SE Darrous L Patxot M. Genetic insights into the age-specific biological mechanisms governing human ovarian aging. Am J Hum Genet. 2023;110:1549–63. 37543033 10.1016/j.ajhg.2023.07.006 PMC10502738

[5] Yagi A Ueda Y Kakuda M. Epidemiologic and clinical analysis of cervical cancer using data from the population-based Osaka Cancer Registry. Cancer Res. 2019;79:1252–9. 30635276 10.1158/0008-5472.CAN-18-3109

[6] Chen G Zhang M Zhu J. Common genetic variants in pre-microRNAs are associated with cervical cancer susceptibility in southern Chinese women. J Cancer. 2020;11:2133–8. 32127940 10.7150/jca.39636 PMC7052933

[7] Eggemann H Ignatov T Geyken CH. Management of elderly women with cervical cancer. J Cancer Res Clin Oncol. 2018;144:961–7. 29500704 10.1007/s00432-018-2617-5 PMC11813448

[8] Nahand JS Taghizadeh-Boroujeni S Karimzadeh M. microRNAs: new prognostic, diagnostic, and therapeutic biomarkers in cervical cancer. J Cell Physiol. 2019;234:17064–99. 30891784 10.1002/jcp.28457

[9] Najafi S. Circular RNAs as emerging players in cervical cancer tumorigenesis; a review to roles and biomarker potentials. Int J Biol Macromol. 2022;206:939–53. 35318084 10.1016/j.ijbiomac.2022.03.103

[10] Volkova LV Pashov AI Omelchuk NN. Cervical carcinoma: oncobiology and biomarkers. Int J Mol Sci. 2021;22:12571. 34830452 10.3390/ijms222212571 PMC8624663

[11] Voutsadakis IA. PI3KCA mutations in uterine cervix carcinoma. J Clin Med. 2021;10:220. 33435133 10.3390/jcm10020220 PMC7827547

[12] Zhang H Wang J Yang J. TMEM33 as a prognostic biomarker of cervical cancer and its correlation with immune infiltration. Mediators Inflamm. 2023;2023:5542181. 37273452 10.1155/2023/5542181 PMC10239303

[13] Rosenthal R McGranahan N Herrero J. DeconstructSigs: delineating mutational processes in single tumors distinguishes DNA repair deficiencies and patterns of carcinoma evolution. Genome Biol. 2016;17:31. 26899170 10.1186/s13059-016-0893-4 PMC4762164

[14] Quinton RJ DiDomizio A Vittoria MA. Whole-genome doubling confers unique genetic vulnerabilities on tumour cells. Nature. 2021;590:492–7. 33505027 10.1038/s41586-020-03133-3 PMC7889737

[15] Love MI Huber W Anders S. Moderated estimation of fold change and dispersion for RNA-seq data with DESeq2. Genome Biol. 2014;15:550. 25516281 10.1186/s13059-014-0550-8 PMC4302049

[16] Subramanian A Tamayo P Mootha VK. Gene set enrichment analysis: a knowledge-based approach for interpreting genome-wide expression profiles. Proc Natl Acad Sci U S A. 2005;102:15545–50. 16199517 10.1073/pnas.0506580102 PMC1239896

[17] Zeng D Ye Z Shen R. IOBR: multi-omics immuno-oncology biological research to decode tumor microenvironment and signatures. Front Immunol. 2021;12:687975. 34276676 10.3389/fimmu.2021.687975 PMC8283787

[18] Newman AM Liu CL Green MR. Robust enumeration of cell subsets from tissue expression profiles. Nat Methods. 2015;12:453–7. 25822800 10.1038/nmeth.3337 PMC4739640

[19] Becht E Giraldo NA Lacroix L. Estimating the population abundance of tissue-infiltrating immune and stromal cell populations using gene expression. Genome Biol. 2016;17:218. 27765066 10.1186/s13059-016-1070-5 PMC5073889

[20] Pollack IF Finkelstein SD Burnham J. Children's Cancer Group. Age and TP53 mutation frequency in childhood malignant gliomas: results in a multi-institutional cohort. Cancer Res. 2001;61:7404–7. 11606370

[21] Chatsirisupachai K Lagger C de Magalhães JP. Age-associated differences in the cancer molecular landscape. Trends Cancer. 2022;8:962–71. 35811230 10.1016/j.trecan.2022.06.007

[22] Spencer CN McQuade JL Gopalakrishnan V. Age at diagnosis impacts molecular and clinical properties of tumors. Cancer Discov. 2022;12:596. 10.1158/2159-8290.CD-RW2022-013 35064031

[23] Nichols CA Gibson WJ Brown MS. Loss of heterozygosity of essential genes represents a widespread class of potential cancer vulnerabilities. Nat Commun. 2020;11:2517. 32433464 10.1038/s41467-020-16399-y PMC7239950

[24] Krajcovic M Overholtzer M. Mechanisms of ploidy increase in human cancers: a new role for cell cannibalism. Cancer Res. 2012;72:1596–601. 22447569 10.1158/0008-5472.CAN-11-3127 PMC3319989

[25] Matsumoto T Wakefield L Peters A. Proliferative polyploid cells give rise to tumors via ploidy reduction. Nat Commun. 2021;12:646. 33510149 10.1038/s41467-021-20916-y PMC7843634

[26] Was H Borkowska A Olszewska A. Polyploidy formation in cancer cells: how a Trojan horse is born. Semin Cancer Biol. 2022;81:24–36. 33727077 10.1016/j.semcancer.2021.03.003

[27] López S Lim EL Horswell S. TRACERx Consortium. Interplay between whole-genome doubling and the accumulation of deleterious alterations in cancer evolution. Nat Genet. 2020;52:283–93. 32139907 10.1038/s41588-020-0584-7 PMC7116784

[28] Kan Z Ding Y Kim J. Multi-omics profiling of younger Asian breast cancers reveals distinctive molecular signatures. Nat Commun. 2018;9:1725. 29713003 10.1038/s41467-018-04129-4 PMC5928087

[29] Chatsirisupachai K Lesluyes T Paraoan L. An integrative analysis of the age-associated multi-omic landscape across cancers. Nat Commun. 2021;12:2345. 33879792 10.1038/s41467-021-22560-y PMC8058097

[30] Lee W Wang Z Saffern M. Genomic and molecular features distinguish young adult cancer from later-onset cancer. Cell Rep. 2021;37:110005. 34788626 10.1016/j.celrep.2021.110005 PMC8631509

[31] Liu W Xie A Tu C. REX-1 represses RASSF1a and activates the MEK/ERK pathway to promote tumorigenesis in prostate cancer. Mol Cancer Res. 2021;19:1666–75. 34183450 10.1158/1541-7786.MCR-20-0974

[32] Zeng YT Liu XF Yang WT. REX1 promotes EMT-induced cell metastasis by activating the JAK2/STAT3-signaling pathway by targeting SOCS1 in cervical cancer. Oncogene. 2019;38:6940–57. 31409905 10.1038/s41388-019-0906-3

[33] Lee YH Pang SW Poh CL. Distinct functional domains of PNMA5 mediate protein-protein interaction, nuclear localization, and apoptosis signaling in human cancer cells. J Cancer Res Clin Oncol. 2016;142:1967–77. 27424190 10.1007/s00432-016-2205-5 PMC11819373

[34] Huang F Cao Y Wang C. PNMA5 promotes bone metastasis of non-small-cell lung cancer as a target of BMP2 signaling. Front Cell Dev Biol. 2021;9:678931. 34136487 10.3389/fcell.2021.678931 PMC8200676

[35] Moretta A. Natural killer cells and dendritic cells: rendezvous in abused tissues. Nat Rev Immunol. 2002;2:957–64. 12461568 10.1038/nri956

[36] Ferlazzo G Tsang ML Moretta L. Human dendritic cells activate resting natural killer (NK) cells and are recognized via the NKp30 receptor by activated NK cells. J Exp Med. 2002;195:343–51. 11828009 10.1084/jem.20011149 PMC2193591

[37] Peterson EE Barry KC. The natural killer-dendritic cell immune axis in anti-cancer immunity and immunotherapy. Front Immunol. 2020;11:621254. 33613552 10.3389/fimmu.2020.621254 PMC7886798

[38] Subramanian M Kabir AU Barisas D. Conserved angio-immune subtypes of the tumor microenvironment predict response to immune checkpoint blockade therapy. Cell Rep Med. 2023;4:100896. 36630952 10.1016/j.xcrm.2022.100896 PMC9873950

[39] Xu J Lamouille S Derynck R. TGF-beta-induced epithelial to mesenchymal transition. Cell Res. 2009;19:156–72. 19153598 10.1038/cr.2009.5 PMC4720263

[40] Hao Y Baker D Ten Dijke P. TGF-β-mediated epithelial-mesenchymal transition and cancer metastasis. Int J Mol Sci. 2019;20:2767. 31195692 10.3390/ijms20112767 PMC6600375

[41] He WQ Li C. Recent global burden of cervical cancer incidence and mortality, predictors, and temporal trends. Gynecol Oncol. 2021;163:583–92. 34688503 10.1016/j.ygyno.2021.10.075

[42] Lieu CH Golemis EA Serebriiskii IG. Comprehensive genomic landscapes in early and later onset colorectal cancer. Clin Cancer Res. 2019;25:5852–8. 31243121 10.1158/1078-0432.CCR-19-0899 PMC6774873

[43] Gerhauser C Favero F Risch T. Molecular evolution of early-onset prostate cancer identifies molecular risk markers and clinical trajectories. Cancer Cell. 2018;34:996–1011.e8. 30537516 10.1016/j.ccell.2018.10.016 PMC7444093

[44] Iengar P. An analysis of substitution, deletion and insertion mutations in cancer genes. Nucleic Acids Res. 2012;40:6401–13. 22492711 10.1093/nar/gks290 PMC3413105

[45] Turajlic S Litchfield K Xu H. Insertion-and-deletion-derived tumour-specific neoantigens and the immunogenic phenotype: a pan-cancer analysis. Lancet Oncol. 2017;18:1009–21. 28694034 10.1016/S1470-2045(17)30516-8

[46] Chalmers ZR Connelly CF Fabrizio D. Analysis of 100,000 human cancer genomes reveals the landscape of tumor mutational burden. Genome Med. 2017;9:34. 28420421 10.1186/s13073-017-0424-2 PMC5395719

[47] Milholland B Auton A Suh Y. Age-related somatic mutations in the cancer genome. Oncotarget. 2015;6:24627–35. 26384365 10.18632/oncotarget.5685 PMC4694783

[48] Berger NA. Young adult cancer: influence of the obesity pandemic. Obesity (Silver Spring). 2018;26:641–50. 29570247 10.1002/oby.22137 PMC5868416

[49] Anders CK Hsu DS Broadwater G. Young age at diagnosis correlates with worse prognosis and defines a subset of breast cancers with shared patterns of gene expression. J Clin Oncol. 2008;26:3324–30. 18612148 10.1200/JCO.2007.14.2471

[50] Maas HA Kruitwagen RF Lemmens VE. The influence of age and co-morbidity on treatment and prognosis of ovarian cancer: a population-based study. Gynecol Oncol. 2005;97:104–9. 15790445 10.1016/j.ygyno.2004.12.026

